# A Functionally Different Immune Phenotype in Cattle Is Associated With Higher Mastitis Incidence

**DOI:** 10.3389/fimmu.2018.02884

**Published:** 2018-12-06

**Authors:** Karina Lutterberg, Kristina J. H. Kleinwort, Bernhard F. Hobmaier, Stefanie M. Hauck, Stefan Nüske, Armin M. Scholz, Cornelia A. Deeg

**Affiliations:** ^1^Chair of Animal Physiology, Department of Veterinary Sciences, LMU Munich, Munich, Germany; ^2^Research Unit for Protein Science, Helmholtz Zentrum Munich, German Research Center for Environmental Health GmbH, Munich, Germany; ^3^Livestock Center of the Faculty of Veterinary Medicine, LMU Munich, Oberschleißheim, Germany

**Keywords:** bovine neonatal pancytopenia, hyperproliferation, differential immune proteome, Concanavalin A, STAT, T helper pathways, deviant immune phenotype, mastitis

## Abstract

A novel vaccine against bovine viral diarrhea (BVD) induced pathogenic antibody production in 5–10% of BVD-vaccinated cows. Transfer of these antibodies via colostrum caused Bovine neonatal pancytopenia (BNP) in calves, with a lethality rate of 90%. The exact immunological mechanisms behind the onset of BNP are not fully understood to date. To gain further insight into these mechanisms, we analyzed the immune proteome from alloreactive antibody producers (BNP cows) and non-responders. After *in vitro* stimulation of peripheral blood derived lymphocytes (PBL), we detected distinctly deviant expression levels of several master regulators of immune responses in BNP cells, pointing to a changed immune phenotype with severe dysregulation of immune response in BNP cows. Interestingly, we also found this response pattern in 22% of non-BVD-vaccinated cows, indicating a genetic predisposition of this immune deviant (ID) phenotype in cattle. We additionally analyzed the functional correlation of the ID phenotype with 10 health parameters and 6 diseases in a retrospective study over 38 months. The significantly increased prevalence of mastitis among ID cows emphasizes the clinical relevance of this deviant immune response and its potential impact on the ability to fight infections.

## Introduction

Vaccines are the most effective and also affordable disease-prevention tools (1) and maternal antibodies protect the offspring from infections in man (2) and animals (3). In cattle, pre-partum vaccination with various virulence factors of bacterial or viral infectious diseases is a standard procedure which effectively stimulates the production of specific antibodies (4, 5). A novel production technology using an allogeneic cell line (6) and the addition of a highly potent adjuvant to PregSure BVD, a new vaccine against bovine viral diarrhea (BVD), induced fatal immune reactions with production of alloreactive antibodies in 5–10% of vaccinated cows (7–9). These alloantibodies were transmitted to calves, regardless of kin, via the colostrum of these dams (8, 10) and caused bovine neonatal pancytopenia (BNP) (7, 11–13), even years after the vaccine has been taken off the market (14). BNP calves suffered from hemorrhagic diathesis, thrombo and leukocytopenia and bone marrow depletion, which resulted in a deadly bleeding disorder (10, 15, 16). Several authors hypothesized alloimmune reactions against the major histocompatibility complex class I (MHC I) as causal for BNP (17–20). This hypothesis is still controversial since MHC I alloantibodies would be expected to bind all nucleated cells, not only to blood-derived and hematopoietic progenitor cells (12, 16). Also transcriptome analyses provided evidence that MHC I can be excluded as single causal agent for BNP-associated alloantibodies (21). As other authors claimed the possibility of a genetic predisposition of BNP dams for production of BNP inducing alloantibodies (22–24), our studies focused on investigating a general difference in immune responses between PregSure BVD vaccinated control cows and BNP donors. We wanted to analyze if there is evidence for differential expression of master transcription factors of cellular immune response in lymphocyte proteome of controls and BNP dams. Interestingly, we found a markedly different immune phenotype in BNP dams, characterized by significant hyperproliferation of lymphocytes and a deviant immune signaling pathway compared to controls. We were next interested to see if we could find evidence for this immune phenotype in cows that were never vaccinated with PregSure BVD. Further testing showed that this altered immune phenotype was also detectable in 22% of cows from a PregSure BVD unvaccinated cohort. This confirmed its natural, not vaccine-induced, appearance in cattle and gave more evidence for immune deviant (ID) phenotype as a genetic predisposition. In further in-depth experiments we found that Interleukin-2 (IL-2) plays an important role in diverging immune response of ID lymphocytes. In addition to these immune response studies, we analyzed the functional correlation of the ID phenotype with overall 10 health parameters and 6 diseases in a retrospective study over 38 months. Here, a significantly increased mastitis incidence in cows with an ID phenotype became apparent, highlighting the clinical impact of altered immune response.

## Materials and Methods

In this study, peripheral blood derived lymphocytes (PBL) of a total of 132 cows were used. Eleven of these cows had previously been vaccinated with PregSure BVD, of which six did not produce BNP-inducing antibodies (BVD vaccinated control cows) and five showed alloreactive antibody production (BNP dams) resulting in their calves dying from hemorrhagic diathesis with confirmed thrombocytopenia, leukocytopenia and bone marrow depletion. The remaining 121 cows were healthy controls (female, age: 2–10 years, first to eight lactation period) from one dairy farm never vaccinated with PregSure BVD. No experimental animals were killed through this study. Withdrawal of blood was permitted by the local authority Regierung von Oberbayern, Munich, permit no. 55.2-1-54-2532.3-22-12.

### PBL Isolation

Bovine venous blood samples were collected in tubes with heparin sodium 25.000 I.U. Blood was diluted with equal parts of phosphate buffered saline (PBS; 136,9 mM NaCl, 2,6 mM KCl, 1,4 mM KH_2_PO_4_, 8,1 mM Na_2_HPO_4_ × 2H_2_O; pH 7,2) and subsequently layered on density gradient separating solution (Pancoll; PanBiotech, Aidenbach, Germany). After density gradient centrifugation (room temperature, 290 × g, 25 min, brake off), PBL were obtained from intermediate phase. Cells were washed twice in PBS (4°C) and either used immediately or stored at −20°C (lysate) for western blot analyses.

### Proliferation Assays

PBL were seeded in 96-well plates (1 × 10^5^ cells/well) and stimulated for 48 h with either PWM (Pokeweed mitogen), ConA (Concanavalin A) or BanLec (Banana lectin) (Sigma-Aldrich, Taufkirchen, Germany, 5 μg/ml), bovine IL-2 (Bio-Techne, Wiesbaden, Germany, 1 ng/ml), bovine IL-4 or bovine Interferon gamma (IFNγ) (Thermo Fisher Scientific, Bremen, Germany, 3 ng/ml). For the inhibition assays, cells were incubated with STAT3 inhibitor (WP 1,066, Santa Cruz, Heidelberg, Germany, 50 ng/ml) 12 h before stimulation. After 34 h of stimulation, cells were pulsed for 14 h with 0,05 mCi/well [methyl-^3^H]-thymidine (Perkin Elmer, Hamburg, Germany), harvested and counts per minute were measured. Proliferation rate after stimulation was expressed as factor of [^3^H]-thymidine incorporation with respect to the unstimulated cells or after inhibition as percentage to ConA stimulated cells.

### Flow Cytometry Analyses

Staining of 5 × 10^5^ cells per well was performed with anti-bovine cluster of differentiation (CD) 4 (mouse IgG1 monoclonal, Bio-Rad AbD Serotec, Puchheim, Germany; 1:100), anti-bovine CD8 (mouse IgG2a monoclonal, Bio-Rad AbD Serotec; 1:50) and FITC-conjugated anti-bovine IgM (Bio-Rad AbD Serotec, Puchheim, Germany; 1:50) antibodies, diluted in staining buffer (1% BSA + 0,001% NaN_3_ in PBS). Respective secondary antibodies anti-mouse IgG1 FITC and anti-mouse IgG2a FITC (both Santa Cruz Biotechnology; Heidelberg, Germany 1:200) were added. All antibodies were incubated for 30 min on ice. Cells were washed with staining buffer between primary and secondary antibody staining steps (200 × g, 4°C, 1 min). For all stainings with secondary antibodies respective isotype controls were used. Cells were fixed in 1% PFA diluted in staining buffer and stored at 4°C until analysis. On FACS Canto II, measurement of cells was performed with FACS Diva Software (BD Biosciences). Lymphocytes were gated according to forward scatter (cell size) and side scatter (intercellular granularity) properties of cells. Per staining, between 5 × 10^3^ and 1 × 10^4^ cells were measured. Further analysis of flow cytometry data was performed using open source Flowing Software 2.5.1 http://flowingsoftware.btk.fi/ (Perttu Terho, Turku Centre for Biotechnology, Finland).

### Differential Proteome Analyses

PBL (2.2 × 10^7^ cells) of two PregSure BVD vaccinated control cows and two BNP dams were stimulated with PWM and ConA (5 μg/ml) for 48 h. For LC-MS/MS analysis, peptides were separated on a reversed chromatography column (75 μm ID × 15 cm, Acclaim PepMAP 100 C18. 100Å/size, LC Packings, Thermo Fisher Scientific, Bremen, Germany) and the analysis was conducted with an Ultimate 3000 nano-HPLC system (Dionex, Idstein, Germany). Nano-HPLC was connected in a linear quadruple ion trap-Orbitrap (LTQ Orbitrap XL) mass spectrometer (Thermo Fisher Scientific, Bremen, Germany). The mass spectrometer was operated in the data-dependent mode to automatically switch between Orbitrap-MS and LTQ-MS/MS acquisition. Up to 10 most intense ions were selected for fragmentation on the linear ion trap using collision induced dissociation at a target value of 100 ions and subsequently dynamically excluded for 30 s. Elevated spectra were imported into Progenesis software (version 2.5). After comparison and normalization, spectra were exported as Mascot Generic files and searched against the Ensembl bovine database (version 80) with Mascot (Matrix Science, version 2.4.1). Peptide assignment was reimported to Progenesis Software. All unique peptides allocated to a protein were considered for quantification. Only proteins quantified with at least two peptides were included for further analysis.

For hierarchical cluster analyses of normalized protein abundances, arithmetic means of two respective vaccinated control and BNP samples were generated and proteins with similar expression patterns were clustered with Perseus software algorithm (version 1.6.1.1; Computational Systems Biochemistry, Martinsried, Germany) (25). Proteins with a ratio of at least 2-fold in normalized abundance between control and BNP samples were defined as differentially.

### Western Blots

For protein expression analyses with western blots, PBL were first lysed in lysis buffer (9 M Urea, 2 M Thiourea, 65 mM Dithioerythritol, 4% CHAPS). Proteins were then separated by SDS-PAGE on 8% gels (7 μg protein/slot) and blotted semidry onto 8.5 × 6 cm PVDF membranes (GE Healthcare, Freiburg, Germany) and blocked with 4% BSA (1 h). Blots were incubated overnight with respective primary antibodies: rabbit anti-pSTAT1 Tyr701, rabbit anti-pSTAT3 Tyr705 (Cell Signaling, Darmstadt, Germany, 1:500) or mouse anti-beta actin (Sigma, Taufkirchen, Germany, 1:5000). As secondary antibodies, either HRP-coupled goat anti-rabbit IgG (H+L) antibody (Cell Signaling, Darmstadt, Germany, 1:5000) or goat anti-mouse IgG (H+L) antibody (Sigma-Aldrich, Taufkirchen, Germany, 1:5000) were used (1 h). Signals were detected by enhanced chemiluminescence on X-ray film (SUPER-2000G ortho, Fuji; Christiansen, Planegg, Germany). Films were scanned on a transmission scanner and densitometric quantification of Western blot signals was performed using ImageJ software (open source: http://imagej.nih.gov/ij/). Abundances of pSTAT1 and pSTAT3 were subsequently normalized to beta actin.

### Health Parameters

Milk production and health parameters (observed by veterinarians) of 100 non-PregSure BVD vaccinated cattle from one dairy farm were analyzed for 38 months retrospectively. Results were then correlated to immune response data of *in vitro* assays. Seventy-three control cows with low proliferation rate after ConA stimulation and 27 ID cows, which showed a BNP-like hyperproliferation to ConA, were included in this study. Ten health parameters [daily milk yield, average lactation performance (300 days), milk structure (lactose, fat, urea, somatic cell count), fertility parameters (amounts of inseminations, calving-to-conception intervals, medicinal induction of oestrus, ovarian cysts)] and 6 diseases [diseases of musculoskeletal system, claws, digestive tract, respiratory system, and metabolic disorders (ketosis, hypocalcemia)] were analyzed. All parameters were recorded and listed by the same veterinarians and the statistical analysis was performed using the odds ratio and chi-square distribution.

### Statistical Analyses

Data were analyzed in Prism software (GraphPad, version 5.04) with Kolmogorow-Smirnow (KS) test. If KS test was significant (*p* < 0.05; normal distribution), Student's *t*-test was used for statistical analysis, if KS test was not significant (*p* > 0.05; no normal distribution) statistics were performed using Mann-Whitney test. For statistical analysis of health parameters, we used odds ratio and chi-square distribution. Differences were considered statistically significant with the following *p*-values: ^*^p < 0.05, ^**^p < 0.01, ^***^p < 0.001, and ^****^p < 0.0001.

## Results

### Lymphocytes of BNP Dams Show Hyperproliferation After Polyclonal Stimulation *in vitro*

**Figure 1:**
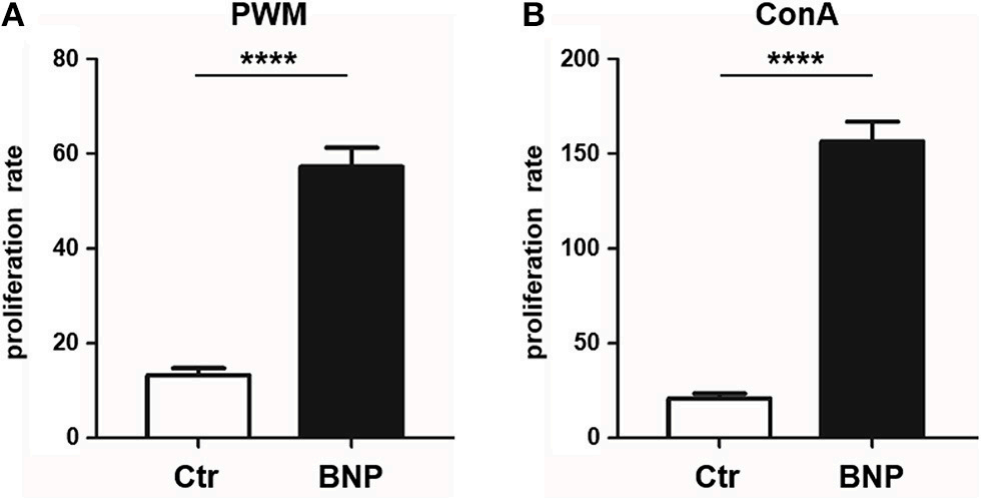
Hyperproliferation of BNP lymphocytes after polyclonal stimulation *in vitro*. **(A)** Lymphocytes from BNP cows (black bars, *n* = 5, technical replicates *n* = 98) proliferated 4.5 times stronger (****p < 0.0001 vs. control) after PWM stimulation than lymphocytes from control dams (white bars, *n* = 6, technical replicates *n* = 53). **(B)** Increased proliferation rate (8-fold; ****p < 0.0001 vs. control) of lymphocytes from BNP dams (black bars, *n* = 5, technical replicates *n* = 86) compared to control dams (white bars, *n* = 6, technical replicates *n* = 52) after *in vitro* stimulation with ConA. Proliferation rate shown in mean ± SD. Mann-Whitney test was used.

After *in vitro* stimulation (48 h) with T and B cell mitogen PWM (26) and T cell mitogen ConA (27), a clearly divergent reaction of lymphocytes from PregSure BVD vaccinated cows became evident. Cells from cows known to produce alloreactive BNP antibodies after vaccination (BNP lymphocytes) proliferated significantly stronger (4.5-fold) than cells from vaccinated control dams after PWM stimulation (Figure 1A, BNP to Ctr, ^****^p < 0.0001) and ConA stimulation (8-fold stronger; Figure 1B, BNP to Ctr, ^****^p < 0.0001). Thus, *in vitro* proliferation assays revealed a highly significant hyperproliferation of BNP lymphocytes demonstrating an increased reaction to polyclonal immune stimulation in these cows.

### No Significant Differences in Lymphocyte Subsets Were Detectable

**Figure 2:**
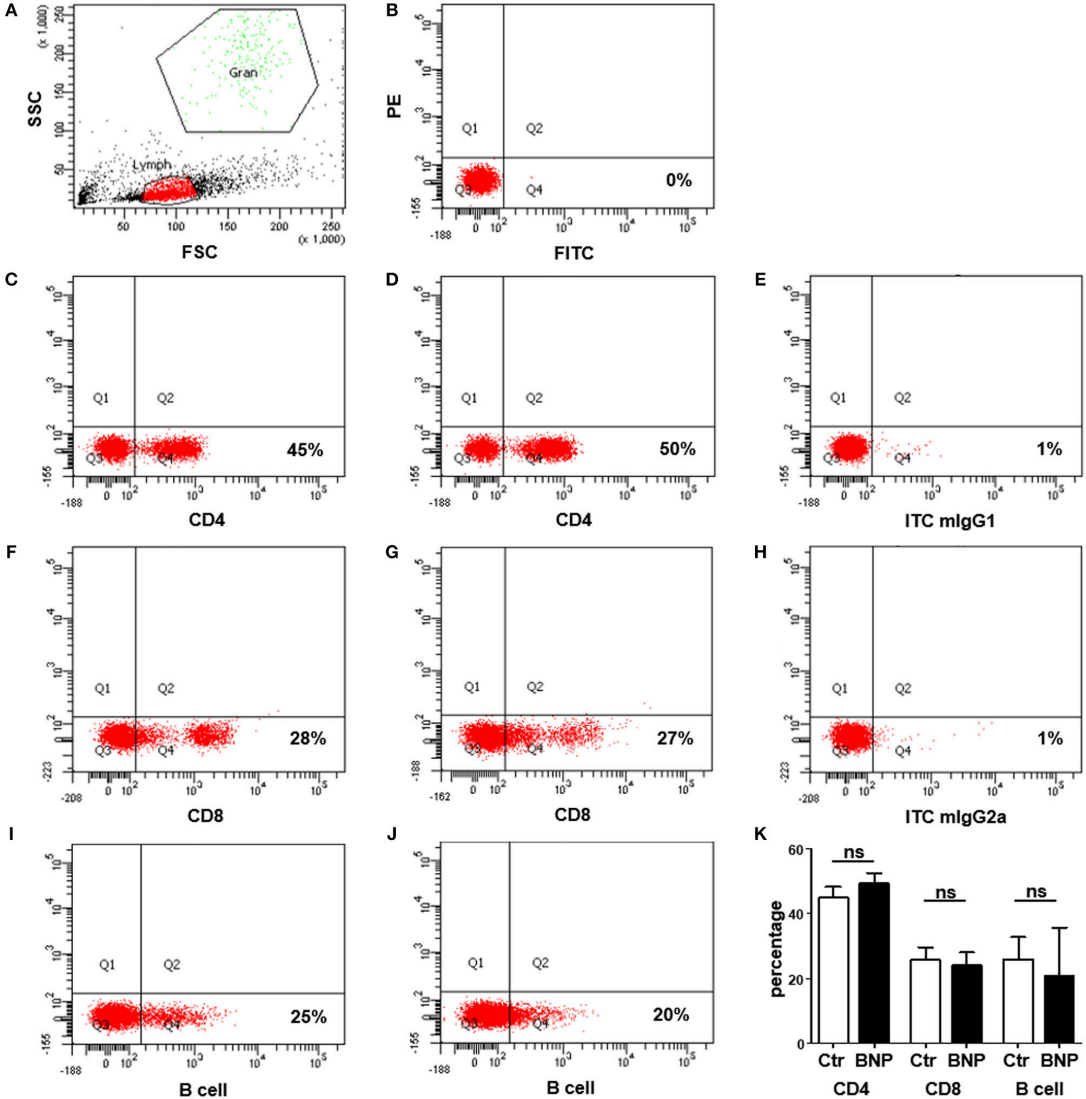
No differences in distribution of lymphocyte subsets between control and BNP dams. **(A–J)** Representative dot plots of flow cytometry analyses from lymphocytes of control and BNP cows. **(A)** Identification of lymphocytes (Lymph) based upon properties of forward (FSC) and sideward scatter (SSC). The population to be measured is shown in red (lymphocytes). **(B)** Unstained cells in Q3 to determine position of quadrants for gating. **(C–J)** Respective staining shown in Q4. **(C)** Expression of CD4 in control lymphocytes (45%). **(D)** Expression of CD4 in BNP lymphocytes (50%). **(E)** Respective isotype control staining for CD4 antibody (mIgG1, 1%). **(F)** Expression of CD8 in control lymphocytes (28%). **(G)** Expression of CD8 in BNP lymphocytes (27%). **(H)** Respective isotype control staining for CD8 antibody (mIgG2a, 1%). **(I)** Expression of B cell marker IgM in control lymphocytes (25%). **(J)** Expression of B cell marker IgM in BNP lymphocytes (20%). **(K)** Flow cytometry analyses of lymphocyte subset distribution revealed no significant differences (ns) between control (white bars; *n* = 4) and BNP cells (black bars; *n* = 3). Percentage of subset is shown in mean ± SD. For statistical analyses Mann-Whitney test was used.

To exclude differences in lymphocyte subset percentages between control and BNP dams as a possible cause for the differential responses toward polyclonal immune stimulation, we analyzed the distribution of CD4^+^, CD8^+^, and B cells in control and BNP PBL. There were no significant differences in proportional composition of subpopulations between PregSure BVD vaccinated control and BNP animals (Figure 2).

### Lymphocyte Proteomes Reveal Differential Protein Regulation Between Normal And Deviant Immune Responders to PregSure BVD Vaccination

**Figure 3:**
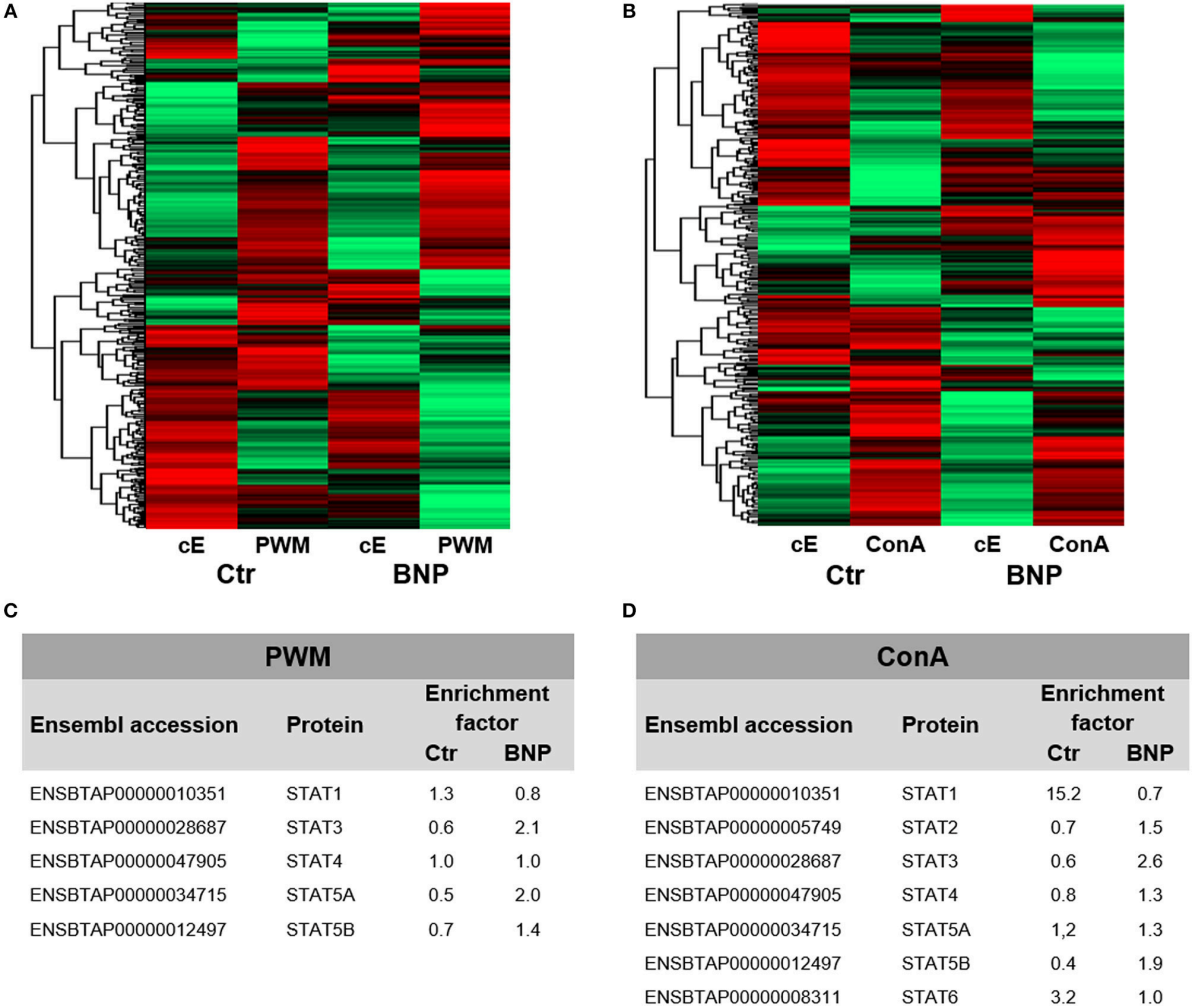
Lymphocyte proteomes reveal regulation of differential expression of proteins between normal and deviant immune responders to PregSure BVD vaccination. Differential proteome analyses of PBL from control (Ctr, *n* = 2, shown as arithmetic means) and BNP (*n* = 2, shown as arithmetic means) cows already revealed differences in protein expression patterns in constitutive expression (cE) of proteins, that shifted even stronger after **(A)** PWM and **(B)** ConA stimulation (48 h). Highly abundant proteins are presented in green and low abundant proteins in red. **(C/D)** In-depth analysis of part of the proteome relating to regulation of immune cells revealed some differentially (factor ≥ 2.0) expressed STAT proteins in bovine PBL after polyclonal activation with PWM **(C)** or ConA **(D)**. STAT3 and STAT5A were selectively upregulated in BNP lymphocytes **(C)** compared to controls due to PWM. After ConA stimulation, controls significantly upregulated STAT1 and STAT6 **(D)**, whereas in PBL of BNP cases STAT3 was enhanced during immune response **(D)**.

To investigate if observed deviant reactions to *in vitro* stimulation originated from differences in immune cell regulation, we executed a differential proteomics experiment with PWM and ConA treated lymphocytes as well as unstimulated cells. Overall, we detected 5,471 proteins. Hierarchical cluster analysis of complete proteomes already visualized fundamental differences in protein abundances between PregSure BVD vaccinated control and BNP cows in constitutive protein levels (cE, Figures 3A,B). Differences in protein expression were also confirmed after immune stimulation (PWM and ConA, Figures 3A,B, hierarchical clustering of all identified proteins). These proteome analyses therefore revealed substantial quantitative differences on protein level.

### Immune Stimulation Leads to Different Usage of STAT Pathways in PBL of Control and BNP Cows

Next, we used our dataset to perform more in-depth analyses on part of the proteome involved in transcription pathways of immune cell regulation. To identify possible differences in master transcription factor expression, we compared lymphocytes from vaccinated control and BNP cows after polyclonal *in vitro* stimulation. Signal transducers and activators of transcription (STAT) induce different T helper (Th) responses in mice and man (24) as master transcription factors. Therefor we analyzed all identified STATs from both proteomic approaches and characterized usage of different STAT pathways as a response to immune stimulation in both cow groups. After polyclonal activation of lymphocytes with PWM, STAT3 and STAT5A were selectively upregulated at least 2-fold in BNP lymphocytes (Figure 3C). ConA stimulation of control cells, however, caused upregulation of STAT1 and STAT6 expression levels (Figure 3D), whereas BNP PBL showed enhanced STAT3 in immune response (Figure 3D). Thus, control and BNP PBL upregulated different master transcription factors as response to stimulation pointing to usage of different immune pathways.

### Lymphocytes of Control and BNP Cows Regulate Different STATs in Response to ConA Stimulation *in vitro*

**Figure 4:**
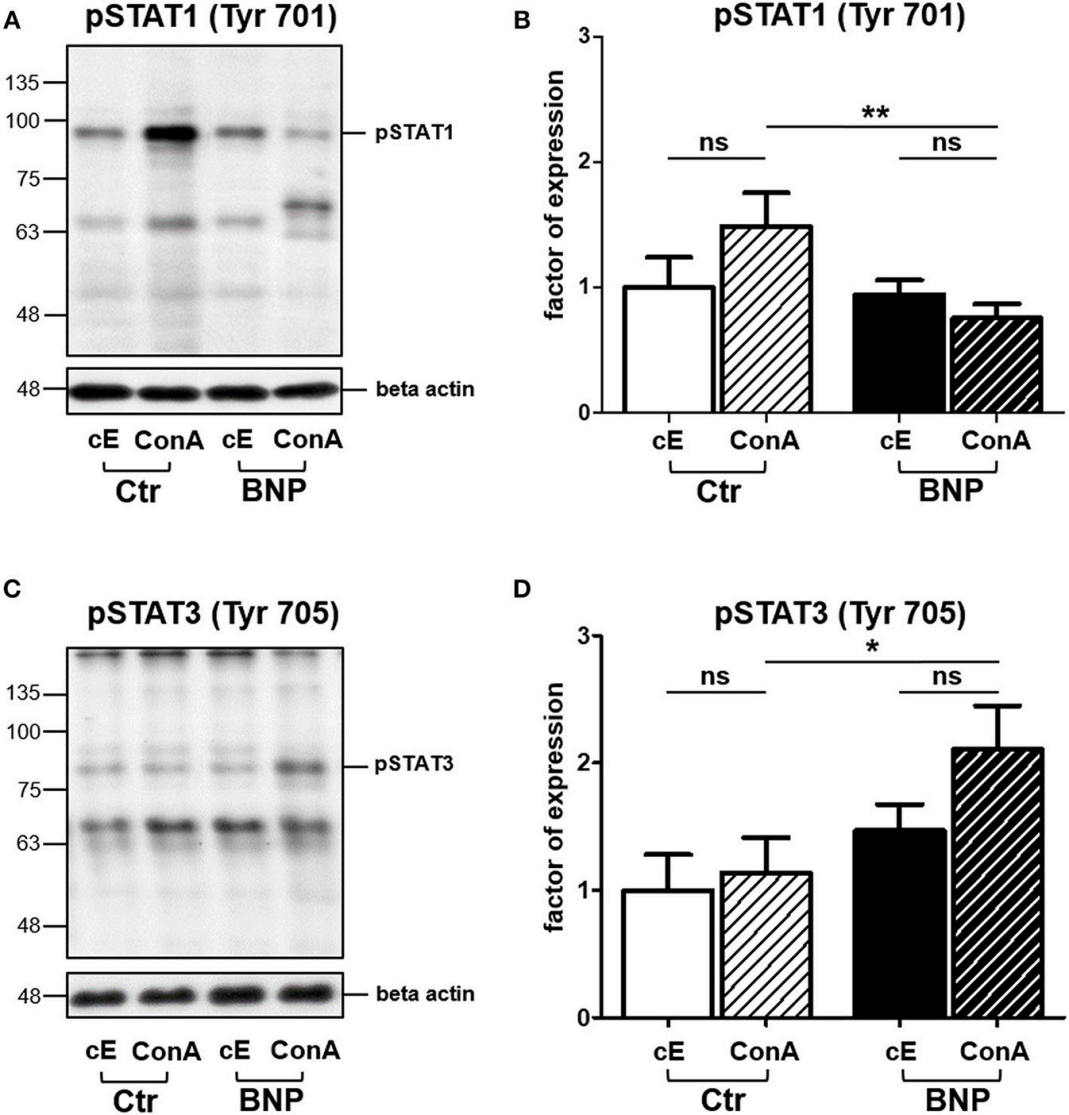
In PBL of control and BNP cows, different transcription factors were activated after ConA stimulation *in vitro*. **(A)** Representative western blot of constitutive (cE) pSTAT1 Tyr701 expression and after stimulation with ConA (ConA) in control PBL (Ctr, *n* = 1) and BNP PBL (BNP, *n* = 1). **(B)** Lymphocytes of vaccinated control cows (white-black striped bars, *n* = 5) phosphorylated STAT1 Tyr701 significantly stronger than BNP lymphocytes (black-white striped bars, *n* = 5) after ConA stimulation (**p < 0.01). **(C)** Representative western blot of constitutive (cE) pSTAT3 Tyr705 expression and after stimulation with ConA (ConA) in control PBL (Ctr, *n* = 1) and BNP PBL (BNP, *n* = 1). **(D)** BNP lymphocytes (black-white striped bars, *n* = 5) phosphorylated STAT3 Tyr705 comparatively stronger than vaccinated control lymphocytes (white-black striped bars, *n* = 5) in response to ConA stimulation (*p < 0.05). **(A/C)** Phosphorylated STAT1 Tyr701 and pSTAT3 Tyr705 signals were normalized to beta-actin and quantified using Image-J software. Protein expression is shown in mean ± SD. For statistical analyses Student‘s *t* test was performed.

Since we decided to further analyze the differential activation of STATs through T cell mitogen ConA, we determined phosphorylation of STAT1 and STAT3 in response to immune stimulation. In lymphocytes of vaccinated controls, phosphorylation of STAT1 Tyr701 significantly increased after *in vitro* stimulation with ConA compared to BNP PBL, where STAT1 Tyr701 phosphorylation decreased after ConA stimulation (Figures 4A,B, Ctr to BNP after ConA stimulation, ^**^p < 0.01). In contrast, lymphocytes of BNP dams phosphorylated STAT3 Tyr705 comparatively stronger than PBL of vaccinated control cows after ConA (Figures 4C,D, BNP to Ctr after ConA stimulation, ^*^p < 0.05). Thus, these experiments ascertained a qualitative difference in immune reactions between the cow groups to a T cell stimulus.

### Inhibition of STAT3 in PBL of BNP Cows Abolishes Hyperproliferation as Reaction to Polyclonal Immune Stimulation

**Figure 5:**
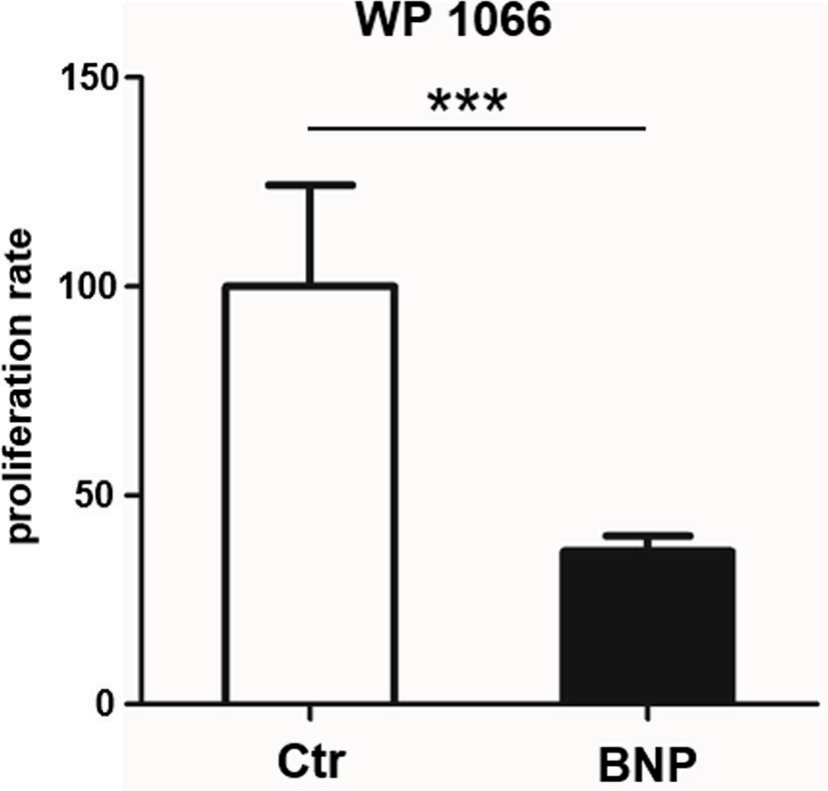
STAT3 inhibitor WP1066 selectively inhibits hyperproliferation of lymphocytes from BNP cows. STAT3 inhibitor did not inhibit proliferation of control PBL (white bar, *n* = 4), but significantly inhibited ConA stimulated BNP cells (black bars, *n* = 4, ***p < 0.001). Percentage of inhibition rate shown in mean ± SD. Student‘s *t-*test was used for statistical analyses.

Since BNP lymphocytes responded to polyclonal stimulation with hyperproliferation and activation of STAT3, we next tested if inhibition of STAT3 reversed respective hyperproliferation. WP1066 (STAT3 inhibitor) significantly reduced proliferation of BNP lymphocytes after ConA stimulation (Figure 5, inhibition of BNP compared to Ctr, ^***^p < 0.001), but not of control lymphocytes. This verified the importance of STAT3-pathway for the hyperproliferation in BNP PBL after polyclonal T cell stimulation.

### Immune Deviant Phenotype Is Also Detectable in Unvaccinated Cows

**Figure 6:**
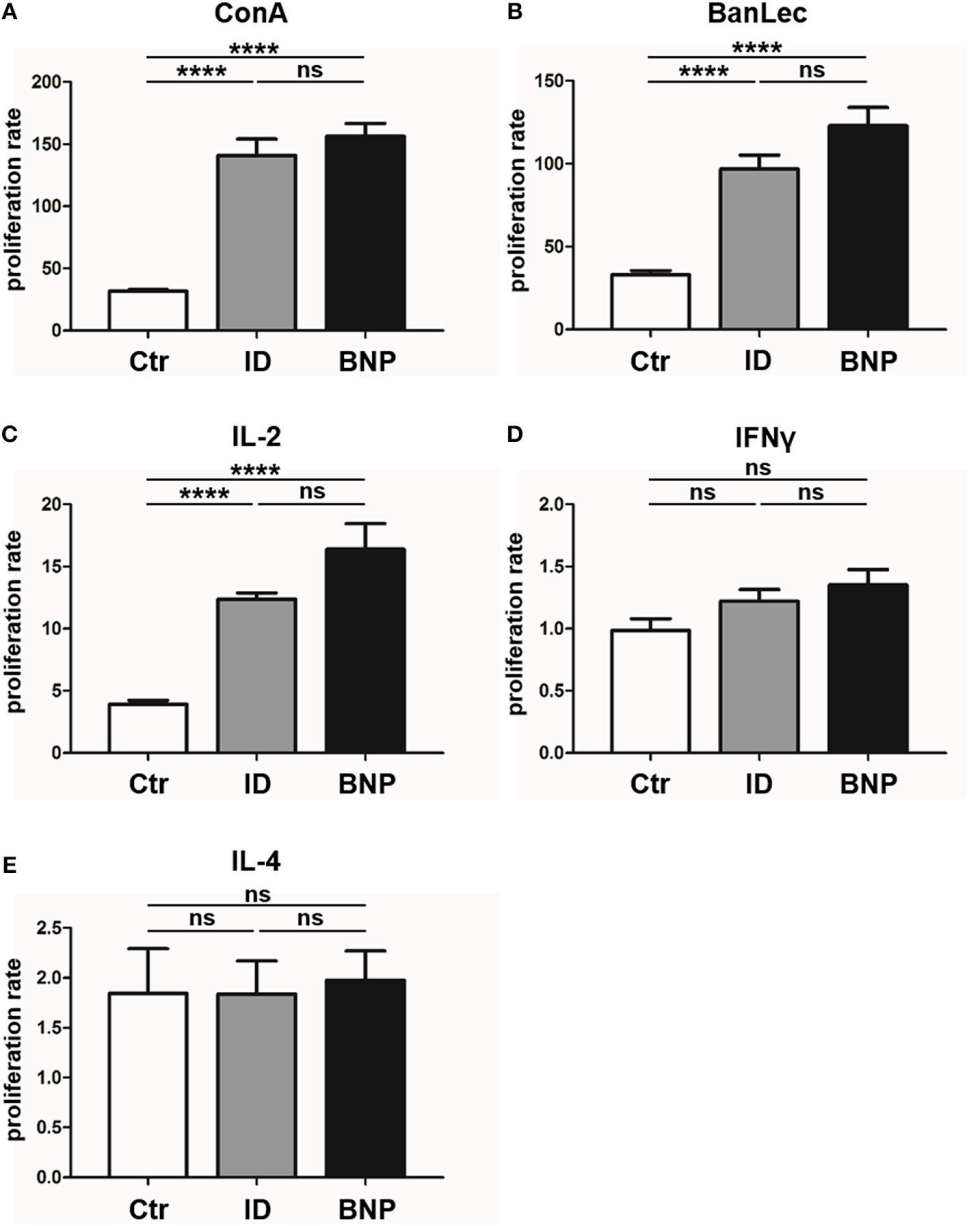
Immune deviant phenotype also detectable in unvaccinated cows. **(A)** In *in vitro* proliferation assays, 22% of unvaccinated cows showed a BNP-like hyperproliferative reaction to ConA [****p < 0.0001 vs. Ctr (white bar, *n* = 121)]. Lymphocytes from ID cows (gray bars, *n* = 27) with a hyperproliferative immune phenotype showed no significant (ns) differences to lymphocytes of BNP cows (black bars, *n* = 4, technical replicates *n* = 86). **(B)** After BanLec stimulation, ID lymphocytes reacted hyperproliferative in comparison to control lymphocytes (****p < 0.0001) and the proliferation rates of these ID lymphocytes did not show significant differences (ns) compared to BNP lymphocytes. **(C)** After IL-2 stimulation, lymphocytes of ID cows also reacted excessively (****p < 0.0001 vs. Ctr) just like lymphocytes from BNP donors (ns). **(D/E)** After bovine IFNγ **(D)** and IL-4 **(E)** stimulation, no significant differences between lymphocytes of unvaccinated and BNP cows were determined (ns). Proliferation rate shown in mean ± SD. Student‘s *t-*test was used.

Since we first detected the deviant immune phenotype in BNP cows, we next tested cows that were never vaccinated with PregSure BVD in order to clarify whether the deviant phenotype was induced through vaccination or if it occurs naturally in a certain subset of cows. Therefore, we examined response of lymphocytes to different polyclonal stimuli in a large group of PregSure BVD unvaccinated cows. To exclude differences caused by environmental factors, cows all came from the same farm. In *in vitro* proliferation assays with T cell mitogen ConA we observed that 22% of the unvaccinated cows reacted similar to BNP dams by showing a hyperproliferative reaction to ConA (immune deviant (ID) phenotype; Figure 6A, reaction difference ID to controls or BNP to controls, ^****^p < 0.0001). Lymphocytes from ID cows with a hyperproliferative immune phenotype did not show significant differences to lymphocytes of BNP cows in these assays (Figure 6A, reaction difference BNP to ID). These data confirmed the natural occurrence of this ID phenotype in cattle and gave stronger evidence for genetic predisposition instead of vaccine-induced appearance.

### Lymphocytes of ID Cows Also React to BanLec and IL-2 With Hyperproliferation

In order to further characterize the pathway targeted by the polyclonal activators PWM and ConA we tested additional mitogens. The T cell mitogen BanLec (28), which activates human T cells through the IL-2 pathway (29, 30), led to similar hyperproliferation of PBL from BNP dams as observed with ConA (Figure 6A, reaction difference factor: 5.0, BNP to controls, ^****^p < 0.0001). PBL of ID cows responded to BanLec stimulation with a similar immune response intensity as BNP cows (Figure 6B, reaction difference factor: 3.0, ID compared to controls, ^****^p < 0.0001). The reaction of lymphocytes showed no significant differences between ID and BNP cows. Since the results with ConA and BanLec pointed to a response via IL-2 pathway, we next tested stimulation with purified bovine IL-2 only. After IL-2 stimulation, lymphocytes of ID cows also reacted excessively (Figure 6C, reaction difference factor: 3.1, ID to controls, ^****^p < 0.0001) and did not show significant differences to BNP lymphocytes (Figure 6C, reaction difference factor: 3.7, BNP to controls, ^****^p < 0.0001).

### IL-2, but Not IFNγ or IL-4 Promote the Different Immune Response in ID Cows

Finally, we tested further signature cytokines for different Th immune responses, bovine Interferon gamma (IFNγ; Th1) and Interleukin 4 (IL-4; Th2), to analyze differentiation of T helper subsets. We detected no significant differences between lymphocytes of vaccinated and unvaccinated controls and BNP cases after IFNγ or IL-4 stimulation *in vitro* (Figures 6D,E). This proves a crucial role for IL-2 in the deviant immune responses, but not for IFNγ or IL-4.

### Immune Deviant Phenotype in Cows Correlates With Significantly Increased Incidence of Mastitis

**Figure 7:**
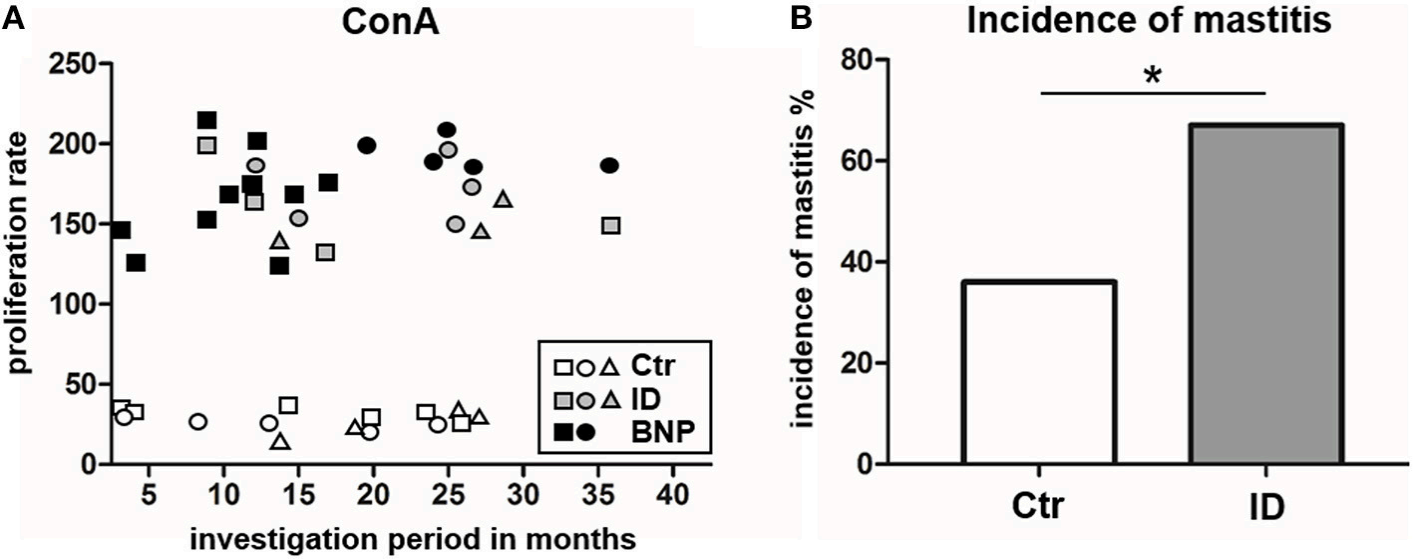
Proliferation response types remain consistent and ID have a significantly increased incidence of mastitis. **(A)** The hyperproliferative phenotype of BNP cows (black) and ID cows (gray) as well as the low proliferation of control cows (white) were consistently detectable over the entire observation period (38 months). Three representative control cows and ID cows are shown and two BNP cows, white symbols = controls, gray symbols = ID, black symbols = BNP. **(B)** ID cows (gray, *n* = 27) suffered twice as often (difference in incidence *p < 0.05) from mastitis than the control cows (white, *n* = 73). Incidence of mastitis shown in percent. Odds ratio and chi-square distribution was used for statistical analyses.

In this study, the proliferative phenotype of controls and ID cows could be repeatedly tested during an overall observation period of 38 months. All of the sampled immune deviant cows showed hyperproliferative reaction to ConA at least three times. Overall, two-thirds of the ID animals consistently showed significant differences in their immune response compared to the unvaccinated control cows (Figure 7A). Since we wanted to know if this newly discovered immune deviant phenotype related to impaired health parameters in respective cows, we retrospectively analyzed milk production, health parameters and different diseases of control and ID cows from the same farm never vaccinated with PregSure BVD. From a total of 16 parameters tested, there was no difference in 15 parameters. However, mastitis incidence was significantly different between both cow groups. We detected that ID cows suffered from mastitis twice as often as control cows (Figure 7B, ^*^p < 0.05). At the moment of blood sampling, all cows were clinically healthy and showed no signs of mastitis which could potentially influence adverse effects to immune stimulation. Thus, this clearly indicates that the hyperproliferative phenotype of ID cows has clinical relevance apart from the response to the BVD vaccine.

## Discussion

BNP was the unwanted result of a fatal immune response to vaccination with PregSure BVD (8, 9). Five to ten percentage of vaccinated cattle produced disease inducing antibodies which were transferred via colostrum, killing 90% of receiving calves, regardless of kin (15, 31). It is still unclear, why a certain subgroup of cows responded differently to the vaccination. We hypothesize that the origin of this altered immune response lies in the existence of a naturally occurring deviant immune phenotype. In this study, we could effectively confirm a quantitative difference in immune cell activation of BNP PBL through hyperproliferative responses *in vitro* (Figures 1A,B). A shift in lymphocyte subpopulations (CD4^+^, CD8^+^, B cells) as a potential reason for these differential responses toward polyclonal immune stimulation could be excluded (Figure 2). The hierarchical clustering of identified lymphocyte proteins from our shotgun proteomics experiment substantiated a general quantitative difference in constitutive expression of proteins in PBL of controls and BNP dams (cE, Figures 3A,B). After immune stimulation, these differences increased even further (PWM/ConA, Figures 3A,B). In order to perform more in-depth investigations of potential functional differences, we subsequently focused on the analysis of master transcription factors of immune cell regulation from our dataset. As a result, we detected several differentially expressed STATs. STATs initiate the differentiation of various Th cell subsets (24) as first-response master regulators and provide lineage specificity by promoting the differentiation of a given Th cell subset while opposing the differentiation to alternative Th cell subsets (32). This makes them especially interesting to us, since expression of different STATs in controls and BNP specimen indicates divergent immune response pathways in these groups. In control lymphocytes we identified STAT1 with increased expression (Figure 3D) and significantly higher activation (Figures 4A,B) after T cell stimulation with ConA. In mice and man, STAT1 is important for the differentiation of naive CD4^+^ T cells to Th1 cells (33) or to type 1 regulatory (Tr) T cells (34). From these findings, we conclude that after immune stimulation, bovine control lymphocytes use the STAT1 pathway and we hypothesize that control cows react with a Th1 or Tr immune response in polyclonal stimulation assays. The effect of STAT1 inhibition could not be tested, since we found no STAT1 specific inhibitor that was commercially available. In contrast to controls, PBL proteome of BNP dams showed higher expression levels of STAT3 (Figure 3D), with increased phosphorylation (Figures 4C,D) in BNP lymphocytes after immune stimulation. STAT3 could therefore be a possible master regulator for the deviant immune response in BNP dams. In mouse and man, STAT3 induces the development of follicular Th (Tfh), Th17, and Th22 cells (32, 35, 36). Our findings proved a STAT3 pathway dependent immune response of BNP lymphocytes after polyclonal stimulation, but so far it is unknown which Th subsets exactly are regulated by STAT3 in cows. The dependence of the hyperproliferative phenotype of immune deviant PBL on STAT3 was shown by inhibition of respective factor using WP 1,066, a cell permeable tyrphostin analog which blocks the STAT3 pathway through inhibition of Janus kinase 2 (JAK2) protein tyrosine kinase (37). As a result, we observed a significantly reduced proliferation to 37% in BNP PBL, but no effect in control PBL (Figure 5). Our findings therefore show, that the deviant immune response of BNP donors is associated with STAT3/JAK2 pathway. Further studies will be needed to clarify whether immune cells from control cows differentiate to Th1 or Tr subsets and if BNP donor cows develop Tfh, Th17, or Th22 cells after immune stimulation. Hyper-reactivity of BNP PBL and the STAT3 driven differentiation of Tfh cells after immune stimulation are supported by findings of other research groups, describing higher alloantibody levels in BNP cows compared to PregSure BVD vaccinated control dams (22). This group also hypothesized, that differences between BNP and control dams are likely due to genes controlling the quantitative alloantibody response after vaccination (22). To find evidence for a genetic predisposition of this immune deviant BNP phenotype in cows (22, 23), we additionally analyzed the immune phenotypes of 121 non-PregSure BVD vaccinated cows. In our study, 22% of cows (ID cows) showed a BNP-like hyperproliferative response to T cell stimulation *in vitro* (ID, Figures 6A,B). This percentage fits to the findings of Benedictus et al., who hypothesized a heritability of 19% in dams for the development of BNP in the calf (22). This proves the existence of an immune deviant phenotype among a certain subpopulation of cattle, which is not triggered by vaccination with PregSure BVD but occurs naturally. The immune deviant response described here in these ID cows clearly depends on IL-2 (Figure 6C), which we could prove through hyperproliferative response of BNP and ID lymphocytes to stimulation with BanLec (Figure 6B), a mitogen that activates human T cells (28, 30) via the IL-2 pathway (29). For bovine T cells, the specific impact of IL-2 itself was not described so far. In man and mice, however, IL-2 has pleiotropic autocrine or paracrine functions that regulate proliferation of T cells (38). It also plays a key role in promoting development, homeostasis and function of regulatory T cells though IL-2/STAT5 signals (39) and balances expression of different STATs in Th cells of mice (40). Therefore, the association of IL-2 with the immune deviant response in cows is a very interesting finding which merits further in-depth investigations in future studies. To determine whether the newly detected deviant immune phenotype is of clinical relevance, we analyzed 10 clinical parameters and 6 diseases in 100 cows from the sampled dairy farm and compared incidence between controls (*n* = 73) and ID animals (*n* = 27). Interestingly, we found a significantly increased mastitis incidence in ID cows compared to controls (Figure 7B). This highly important correlation points to an altered immune reaction of ID cows to classical mastitis pathogens, facilitating mastitis onset in these animals.

In conclusion, we could prove the existence of an immune deviant phenotype in 22% of cattle, regardless of the vaccination status. The ID phenotype shows altered reactions to immune stimulation identical to BNP dams, such as hyperproliferation of lymphocytes, STAT3/JAK2 regulated pathway and association with IL-2. These features potentially triggered alloantigen-production after maternal PregSure BVD vaccination, causing BNP. The correlation of the ID phenotype with high mastitis incidence (Figure 7B) underlines its clinical relevance.

## Author Contributions

CD conceived and designed the experiments. KL, KK, and BH performed the experiments. KL, KK, SH, SN, AS, and CD analyzed the data. KL, KK, and CD wrote the manuscript. All authors critically read the manuscript and approved the final version to be published.

### Conflict of Interest Statement

The authors declare that the research was conducted in the absence of any commercial or financial relationships that could be construed as a potential conflict of interest. The reviewer VR and handling Editor declared their shared affiliation at the time of review.

## Abbreviations

BanLec: Banana lectin
BNP: Bovine neonatal pancytopenia
ConA: Concanavalin A
CD: Cluster of differentiation
ID: Immundeviant phenotype
IFNγ: Interferon gamma
IL: Interleukin
JAK: Janus kinase
MHC I: major histocompatibility complex class I
PBL: peripheral blood lymphocytes
PWM: Pokeweed Mitogen
STAT: Signal transducer and activator of transcription
Tfh: follicular T helper cells
Th: T helper cells
Tr: regulatory T helper cells.
